# Mapping safety in space: the emerging role of spatial transcriptomics in safe drug development

**DOI:** 10.3389/ftox.2026.1817521

**Published:** 2026-06-29

**Authors:** Athena Golfinos-Owens, Terry Van Vleet, Rebecca Kohnken, Prathap Kumar Mahalingaiah, Stacey Fossey, Wayne Buck, Wei Liang, Shuaib Ali Khan Mayana, Anik Tuladhar, Panuwat Trairatphisan, Andy Vo

**Affiliations:** 1 Investigative Toxicology and Pathology, AbbVie, Lake County, IL, United States; 2 Clinical Pharmacology and Safety Sciences, AstraZeneca, Gaithersburg, MD, United States; 3 Preclinical Safety, Sanofi, Frankfurt, Germany

**Keywords:** drug development, preclinical safety, single-cell RNA-seq, spatial omics, spatial transcriptomics, toxicogenomics, toxicology

## Abstract

Bulk tissue transcriptional profiling has played a key role in drug discovery and preclinical safety assessment by revealing mechanisms of toxicity. However, analyzing gene expression from whole tissues is challenging when toxicities are restricted to specific cell types or regions. Newer sequencing methods, including single-cell RNA sequencing and spatial transcriptomics, enable researchers to study cellular and spatial responses at higher resolution. These techniques provide unique insights into localized gene expression changes linked to drug toxicity. Despite their promise, spatial methods are not always the most practical option, as they present challenges related to cost, data complexity, and the need for specialized analytical expertise. Selection of an appropriate platform may depend on study objectives, tissue and species context, and the distribution of affected regions. Thoroughly assessing each platform’s analytical strengths, resolution, and practical limitations is crucial for effectively applying spatial transcriptomics in preclinical safety research. In this review, we evaluate spatial transcriptomic approaches to inform omics platform selection for preclinical safety studies, highlighting platform-specific strengths and considerations specific to drug development.

## 1 Introduction

For more than 2 decades, bulk tissue transcriptional profiling has been a cornerstone of drug safety and development. Its use in preclinical toxicology is widespread, with many experts in the pharmaceutical industry calling it “game changing” for investigative toxicology (Pognan et al., 2023). The concept of toxicogenomics, applying gene expression analysis to assess safety at relevant doses, started with microarray technology and has since evolved to include a wider range of omics technologies (Kramer and Kolaja, 2002). Recent advances in RNA sequencing (RNA-seq) have enhanced this approach, allowing for more accurate gene identification, broader detection of transcripts, and improved quantification (Rao et al., 2018). Traditionally, toxicogenomics has focused on three main areas: uncovering mechanisms of toxicity, prioritizing drug candidates, and predicting safety outcomes using transcriptional profiles (Kramer and Kolaja, 2002). Early success was expected in mechanistic studies, but the hope was that robust toxicity prediction would also become possible over time.

In practice, toxicogenomics is especially useful for generating new hypotheses and quickly clarifying how toxicities arise (Blomme et al., 2009). Numerous studies have shown how these methods can reveal mechanisms of toxicity (Chen et al., 2012; Davis et al., 2015; Marin-Kuan et al., 2006), identify potential biomarkers (Chiusolo et al., 2010), aid risk assessment (Dalmas et al., 2011; Waters et al., 2010), and lend understanding to species differences or translatability to humans (McMillian et al., 2004). For predictive studies, researchers mostly rely on databases that compare gene expressions in tissues from animals treated with known toxicants. Collections of toxicity signatures for various compounds have been established to support these efforts (Li et al., 2015; Nie et al., 2006). In addition, toxicogenomic signatures have been collected in a repository for use by investigators (Darde et al., 2018). Toxicogenomic profiling across 33 compounds highlighted how these methods have improved our understanding of toxicity mechanisms and lowered attrition rates (Foster et al., 2007).

Although valuable, predicting toxicity from gene expression patterns has proven more challenging than expected (Yang et al., 2004), as highlighted by a survey of 13 pharmaceutical companies (Vahle et al., 2018). Bulk RNA-seq analyzes whole tissue to provide average gene expression, but tissue heterogeneity restricts its ability to pinpoint regions or hotspots of toxicity. As each cell type within the organ or tissue has a unique molecular profile shaped by its environment and neighboring cells, incorporating single-cell and spatial context is important for improving toxicogenomic sensitivity. Early spatial technologies such as laser capture microdissection (LCM) (Matsushita et al., 2018; Roberts et al., 2004; Yin et al., 2007), *in situ* hybridization (ISH) and immunohistochemistry (IHC) (Kind, 2000; Monné Rodríguez et al., 2023) have improved mechanistic and spatial understanding of drug-induced toxicity. However, except for LCM, these methods typically focus on specific protein markers, making them better suited for validation studies rather than broad or exploratory analyses in early drug development.

**FIGURE 1:**
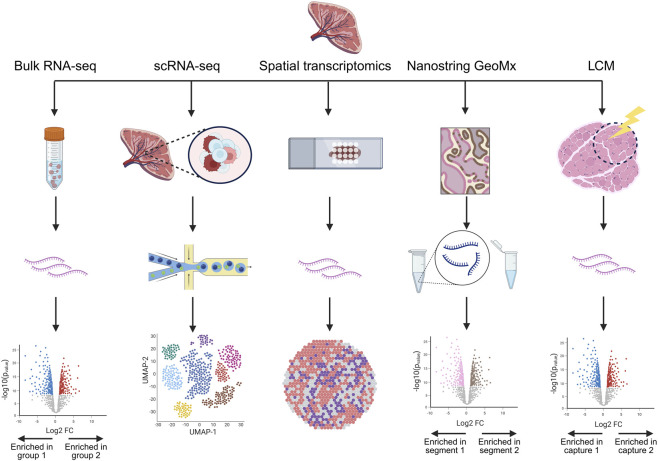
Transcriptomics-based approaches for tissue characterization in safety evaluation studies. All figures were generated on an Enterprise license of Biorender. Publication licenses require listing the journal to be published in and expected date of publication. Publishing licenses will be requested upon manuscript acceptance.

Recent progress in RNA sequencing has introduced single-cell RNA-seq (scRNA-seq) and spatial transcriptomics (ST) (Figure 1). ScRNA-seq enables the identification of individual cell types within a tissue (Chambers et al., 2019) and provides insight into changes in gene expression and cell-to-cell communication, offering more detail and less noise than bulk RNA-seq (Chen et al., 2019; Hebenstreit, 2012; Williams et al., 2022). However, these technological gains bring greater complexity to study design and data analysis (Hebenstreit, 2012; Zhang et al., 2019). While scRNA-seq has improved safety research, it cannot show where different cell types are located, how they interact with each other, or pinpoint areas of toxicity. ST fills this gap by mapping gene expression to exact tissue regions and combining this spatial information with molecular profiles found using scRNA-seq.

Spatial transcriptomics profiling enables mRNA quantification of a number of genes up to the whole transcriptome level, while preserving essential spatial information that deepens our understanding of drug-induced toxicity (Moses and Pachter, 2022). This technology unites some of the most desirable features of other assays into a single experiment: sensitive mRNA detection with up to whole transcriptome coverage, high resolution spatial context that can be directly mapped back to histological findings using H&E, and at times, single cell or subcellular resolution to pinpoint areas of pathological interest such as cellular infiltration or cytokine accumulation. ST allows toxicologists to detect changes in small cell populations, and more accurately pinpoint which cells or tissue zones are affected by drug exposure (Cao et al., 2024). Since drugs can distribute differently depending on their molecular properties and their administration route, ST can be an effective way to clarify these patterns when methods such as ISH, IHC, LCM, and scRNA-seq do not offer sufficient granularity for the given research question. By integrating ST data with histopathology and drug or metabolite measurements using tools like imaging mass spectrometry (Franzén et al., 2024; Kashimura et al., 2018; Sahin et al., 2020; Yang J. et al., 2025), researchers can relate molecular changes to tissue structure, differentiate between primary and secondary effects, and visualize how drugs and metabolites are distributed. Distinguishing spatial patterns between normal and lesioned tissue across time and dose in safety studies can provide insights into lesion progression and adverse effects following drug exposure in a dose-dependent manner.

ST offers the potential of improved insight into gene expression changes linked to drug-induced toxicity while maintaining detailed spatial context, yet its adoption is often limited by high costs, increased data storage needs, longer analysis times, and varying levels of platform maturity (Alexandrov et al., 2023; Atta and Fan, 2021; Choe et al., 2023; Fang et al., 2023). These limitations are further exacerbated in the context of drug development timelines and the need to evaluate multiple conditions (e.g., duration, dosages, species, *etc.*). As spatial technologies become more widely available, choosing the right methods will be important for accurate screenings in drug safety. This review examines the growing role of spatial technologies in pharmaceutical drug safety, highlighting their advantages and limitations within preclinical safety.

## 2 Spatial transcriptomic technologies

Spatial omics platforms have revolutionized the ability to profile gene and protein expression while preserving spatial context across a wide variety of input tissues relevant to safety studies. When both deep sequencing across all or most of the transcriptome and spatial organization is essential context, this emerging technology provides significantly improved granularity compared to other omics technologies. However, selecting a study takes careful consideration due to the wide variety and diversity of spatial platforms made to accommodate a variety of research questions. Broadly, some platforms prioritize subcellular localization and limit gene panels to hundreds or a couple thousand genes, whereas others prioritize whole transcriptome coverage but offer less detailed spatial context. Even more platforms fall somewhere between these two extremes. In the following section, we detail the most common spatial technologies and platforms, highlighting key features from each that can help define their best use cases.

**FIGURE 2:**
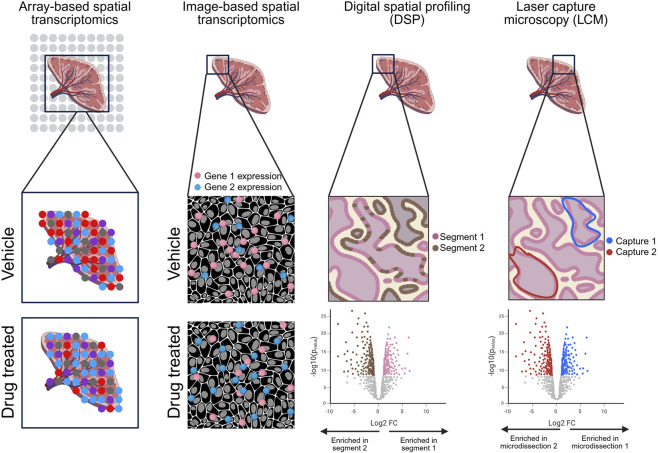
Comparative workflows of major spatial omics technologies, including array-based and image-based spatial transcriptomics, digital spatial profiling, and laser capture microscopy. All figures were generated on an Enterprise license of Biorender. Publication licenses require listing the journal to be published in and expected date of publication. Publishing licenses will be requested upon manuscript acceptance.

There are multiple transcriptomic methods including ST available to toxicologists and pathologists to answer safety-related questions (Figures 1, 2). Depending on whether the study is exploratory or targeted in nature, technologies can be selected by considering their inherent advantages and disadvantages (Table 1). ST platforms are generally classified as either image-based or array-based (Cheng et al., 2023; Dries et al., 2021; Jeon et al., 2023; Liu et al., 2021; Moor and Itzkovitz, 2017; Moses and Pachter, 2022; Park et al., 2023; Rao et al., 2021; Williams et al., 2022) (Figure 2). Array-based technologies use sequencing methods to capture transcriptomic data across tissue sections. In contrast, image-based platforms use high-resolution imaging to visualize and localize transcript expression within tissues. This distinction influences each platform’s analytical capabilities, spatial resolution, and the type of data generated for downstream applications. Advantages to array-based platforms for toxicity studies include whole transcriptome sequencing and the ability to profile larger sections of tissue than many image-based technologies, enabling unbiased spatial discovery but with moderate resolution compared to many imaging-based technologies. Namely, array-based methods typically lack subcellular or single-cell resolution (Cheng et al., 2023; Liu et al., 2021; Moses and Pachter, 2022; Park et al., 2023; Rao et al., 2021; Williams et al., 2022). While it is common to use a scRNA-seq reference atlas to infer single-cell resolution in bulk array-based ST methods, this may not be as robust as image-based ST that leverages higher sensitivity and, in some cases, subcellular resolution. New ST technologies help overcome these previous limitations. For example, 10x Genomics Visium HD offers improved spatial accuracy and can resolve gene expression at the single-cell level (Oliveira et al., 2025). Xenium, an imaging-based ST platform also from 10x Genomics, provides subcellular resolution and enables multiomic profiling (“Xenium In Situ Platform,” 2024). These capabilities go beyond what was possible with earlier platforms like Visium V1 and Visium HD, which did not offer subcellular resolution or multiomic profiling.

**TABLE 1:** Key features of widely-adopted spatial profiling methods.

Technology	Description	Target type	Coverage	Throughput	Input type	Key strengths	Key limitations
Array-based ST (e.g., 10x Visium)	Barcoded spatial capture array with RNA-seq	RNA	Whole-transcriptome (WT)	Moderate–high	Frozen, FFPE	Unbiased WT view; simple workflow	Computationally expensive; single-cell resolution limited
Image-based ST (e.g., MERFISH, CosMx)	Multiplexed fluorescent *in situ* imaging of transcripts	RNA	Targeted gene panels	Moderate-high	Frozen, FFPE	Subcellular/single-cell spatial resolution	Preparation intensive; limited gene panels
GeoMx DSP (Nanostring)	Spatial/molecular profiling with oligo-tagged probes	RNA, protein	Targeted panels or WT	Moderate	FFPE, frozen	Flexible ROI selection; protein + RNA multiplexing	Needs specialized equipment; time-consuming setup
LCM	Microscopy-isolated regions for sequencing	RNA, protein	WT (bulk)	Low	Frozen (preferred); FFPE	Precise region selection; good morphological context	Low throughput; labor intensive; low FFPE RNA yield
IHC	Antibody-based protein detection	Protein	Classic: 1–3 markers; multiplexed: 20-60	Low–moderate	FFPE, frozen	Widely used; robust protein localization	Limited multiplexing; antibody validation needed
ISH	Hybridization-based RNA localization	RNA	Few targets; HiPlex: ∼12–48	Low–moderate	FFPE, frozen	High signal-to-noise and spatial accuracy	Limited multiplexing; low throughput

In contrast to array-based ST, image-based ST often enables targeted tissue visualization at subcellular resolution. Image segmentation methods are used to visualize transcript localization in individual cells, allowing classification of cell types and states. These assays usually profile smaller areas than array-based ST and often focus on a targeted panel of genes. However, many companies offer ways to design and add custom gene panels. These methods are best combined with a defined hypothesis and a specific set of genes for targeted safety inquiry. One practical consideration with imaging-based techniques is that they often require more time and resources than other array-based methods, in part due to the potential need for antibody testing and optimization for custom panels, acquisition and storage of large amounts of imaging data, and identification of an effective segmentation method.

Array- and image-based platforms make up most ST technologies, but there are related methods that can offer spatial information at a lower resolution (Figure 2). These methods include LCM, ISH, IHC, and NanoString GeoMx Digital Spatial Profiler (DSP). LCM uses a laser under microscopic visualization to cut out specific tissue sections for bulk RNA sequencing (Trejo et al., 2019), useful for studying targeted regions, such as lesions identified by pathologists. However, LCM is labor-intensive, has low throughput, and often cannot reliably extract enough RNA from small samples, especially from formalin-fixed, paraffin-embedded (FFPE) tissues. Newer tools like TempO-seq can now help with gene analysis directly from these samples (Trejo et al., 2019; Wijaya et al., 2025). Importantly, LCM only reveals the bulk transcript profile of the selected tissue and does not offer finer transcriptional detail. IHC identifies where specific proteins are expressed in tissue sections. While classic IHC is generally limited to detecting one or two markers, sequential immunostaining methods can identify up to 30 (Bolognesi et al., 2017), though they are time-consuming and require careful antibody optimization and removal between cycles. Fluorescence-based IHC is widely used to study protein co-localization but is restricted by the availability of compatible antibodies from different hosts. Tyramide signal amplification (Lim et al., 2018) can increase the number of detectable proteins beyond what standard fluorescence IHC can achieve. Newer technologies—such as MALDI HiPLEX-IHC (Lim et al., 2023), Phenocycler/CODEX (Heinrich et al., 2022), InSituPlex® (Manesse et al., 2020), multiplexed ion beam imaging (Ptacek et al., 2020), and ChipCytometryTM (Campbell et al., 2023)—now allow detection of more than 30 markers at once. However, all these methods still require *a priori* knowledge of the proteins to be profiled. ISH, like IHC, maps the location of specific genes and RNA in tissue sections. While ISH typically only allows detection of a few targets at once, newer HiPlex RNAScope assays can identify up to 12 targets in FFPE tissues and up to 48 in frozen or fixed frozen samples. When combined with IHC, especially RNA-ISH, both spatial gene and protein information can be collected, but may be limited by target and antibody availability (Ball et al., 2023; Marano et al., 2023; Millar, 2020).

NanoString GeoMx DSP and similar platforms profile tissue microenvironment heterogeneity within chosen regions of interest (ROIs) (Eddy et al., 2024; Glyn et al., 2024; Goralski et al., 2024). Individual ROIs can be further dissected using segmentation or geometric/contour profiling. This approach is useful for comparing transcriptionally or morphologically distinct segments, such as those with high *versus* low levels of a target gene or comparing segments each expressing high levels of a different gene (Goralski et al., 2024). DSP also allows target customization and access to various transcriptome atlases. It also often requires less computational power than other omics methods, as analysis is often similar to bulk RNA-seq. However, spatial resolution of DSP is lower than array- and image-based ST, as each ROI segment typically includes at least 50 cells for protein and 100 cells for RNA (Bergholtz et al., 2021).

**FIGURE 3:**
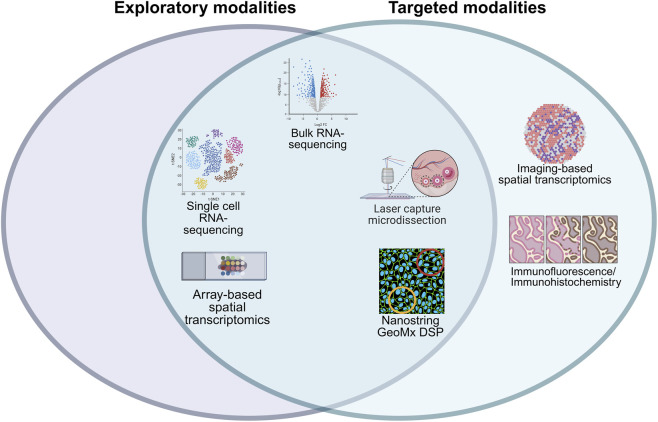
Decision framework for spatial omics platform selection based on study objectives—exploratory *versus* targeted modalities. All figures were generated on an Enterprise license of Biorender. Publication licenses require listing the journal to be published in and expected date of publication. Publishing licenses will be requested upon manuscript acceptance.

Selecting an appropriate spatial platform requires a systematic evaluation of the types of questions that can be addressed with each of these technologies (Table 2). The nature of the research objective, whether exploratory or hypothesis-driven, can further inform platform selection (Figure 3). However, it should be noted that most technologies demonstrate some flexibility to address both exploratory and targeted research questions, with the specific research aims ultimately determining their suitability. Examining prior research that has applied spatial platforms to safety assessments can offer valuable insights into platform selection and help determine which methods are most compatible with a particular research question.

**TABLE 2:** Suggested best-use cases for omics technologies by type of research question.

Method	Exploratory studies	Targeted studies	Primary output data	Example toxicology research question
Imaging/staining (IHC/immunofluorescence)	No	Yes	Imaging of a small number of markers in tissue	Where is CD3 expressed in the tissue?
Bulk RNA-seq	Yes	Yes	Gene by sample matrix	Which genes are upregulated in treatment compared to vehicle?
LCM	Limited	Yes	Gene by sample matrix per region	Which genes are expressed in lesioned vs. non-lesioned regions?
NanoString GeoMx DSP	Limited	Yes	Gene by ROI matrix	
Single-cell RNA-sequencing	Yes	Limited	Gene by cell matrix per sample	Which cell populations change in proportion with increasing drug dose?
Array-based spatial omics	Yes	Limited	Gene by spot/observation matrix with coordinates	What tissue regions show toxicity after drug treatment?
Image-based spatial omics	No	Yes	Gene/protein intensity per cell/region with spatial coordinates	Which cells within a specific region express toxicity-related genes or markers?

## 3 Applications of spatial transcriptomics in toxicology

Applied spatial profiling approaches provide a mechanistically rich framework for evaluating drug safety by profiling molecular changes in the context of tissue architecture, which preserves critical positional information and injury microenvironments. These benefits enable toxicologists to localize early molecular perturbations to specific functional compartments, connect those perturbations to evolving lesions, and prioritize region-specific biomarkers and pathways (Wu et al., 2021; Zhang L. et al., 2022). Spatial omics are increasingly used to explore toxicology to (1) resolve compartment-restricted responses, (2) characterize microenvironments that coordinate injury, inflammation, and repair, (3) quantify spatially patterned dose responses, and (4) integrate molecular readouts for stronger weight-of-evidence decisions.

The liver is most frequently highlighted in safety-related spatial profiling studies because hepatotoxicity is a significant concern to drug development. It requires screening in toxicology studies and can manifest patterns organized along hepatic lobular zones with distinct functions (Thakkar et al., 2020). Spatial profiling has clarified how different insults may impact centrilobular, midzonal, or periportal regions, and ultimately drive necrosis, immune infiltration, and regeneration. For example, spatial analysis of ischemia–reperfusion (I/R) injury identified pericentral regions as particularly vulnerable and linked these signatures to spatially restricted immune and hepatocyte injury programs when interpreted alongside histopathology (Xin et al., 2023). Importantly, linking spatial signatures to established perturbational signature databases like the Connectivity Map enables researchers to identify connections with known drug signatures, such as Celastrol, a candidate modulator of HIF1α-associated injury programs (Lamb et al., 2006; Xin et al., 2023). Following acetaminophen exposure, spatial studies have detected early zone-dependent transcriptional changes consistent with differential detoxification capacity and stress responses across lobular compartments, capturing molecular features that can be subtle or diluted in non-spatial assays (Cao et al., 2024; Umbaugh et al., 2021). Spatial omics also provides a direct window into how parenchymal responses are shaped by non-parenchymal compartments. In murine acetaminophen injury, integration of ST with scRNA-seq differentiated zone-specific programs involving hepatocytes, Kupffer cells, and endothelial cells during regeneration, helping to distinguish localized repair niches from broader inflammatory activation (Ben-Moshe et al., 2022).

Beyond acute injury, spatial approaches are increasingly informative for pathway-level disruption of liver organization that is central to chronic or dose-dependent hepatotoxic mechanisms. In a study of 2,3,7,8-tetrachlorodibenzo-p-dioxin exposure, spatial profiling revealed dose-dependent remodeling of zonation with loss of portal identity across the lobule, providing a tissue-resolved view of how endocrine and xenobiotic signaling may reprogram hepatic patterning (Nault et al., 2023). Additional work in diet and chemical injury have also linked spatially restricted metabolic effects to disease-relevant phenotypes. For instance, combined Western diet and carbon tetrachloride exposure induced periportal enrichment of CYP2E1-associated and lipid metabolism genes that recapitulate features relevant to pediatric fatty liver disease biology in mouse models (Jiang et al., 2024). Consistent with these observations, ectopic expression of Wnt modulators such as RSPO3 across lobular regions has been reported to disrupt canonical zonation (Zhou et al., 2024). Collectively, these liver studies illustrate potential applications of spatial profiling for safety assessment in drug development. Spatial methods can identify where injury-associated programs reside, how they spread across functional areas, and which pathways could be targeted or monitored as early indicators of adverse outcomes.

The kidney represents a parallel, and in some respects more demanding, use-case because nephrotoxicity often manifests as a compartment-specific injury within a highly segmented organ. Tubular segments, glomeruli, and interstitial regions exhibit distinct baseline transcriptomes and injury susceptibilities that can be difficult to deconvolute without spatial context (Balzer et al., 2022). Spatially and temporally resolved profiling of acute kidney injury has been used to track region-specific lesion development, evolving cell–cell interactions, and molecular transitions toward maladaptive repair and fibrosis, enabling a more granular mapping between transcriptomic state and pathologic stage (Dixon et al., 2022). In a rat model of losartan-associated findings, integration of ST with single-cell data resolved heterogeneity in glomerular responses and supported renin pathway activation consistent with hypotensive feedback physiology, highlighting how spatial context can clarify whether observed signals reflect direct toxicity, compensatory adaptation, or both (Onoda et al., 2022). Complementary work combining spatial and single-cell approaches following endotoxin exposure has identified distinct transcriptional programs across renal tubular epithelial segments, which would be difficult to assess with more traditional methods (Janosevic et al., 2021). Importantly for safety assessments, methodologies beyond array-based platforms can add resolution in anatomically complex tissues. For example, LCM coupled with targeted sequencing has been applied to nephron subregions in rats to identify region-specific DNA damage signaling, inflammatory activation, actin cytoskeletal remodeling, and metabolic reprogramming associated with cisplatin treatment (Wijaya et al., 2025).

Although liver and kidney are most frequently seen in literature as current toxicology-relevant examples, the same conceptual advantages extend to other organ systems where adverse findings are spatially patterned or regionally constrained. In the central nervous system, spatial profiling has been used to localize transcriptional changes to specific neuroanatomic regions, such as thalamic and ventricular areas, where pathway perturbations relevant to neurogenesis and myelination processes can be assessed in an explicitly regional manner (Xu et al., 2024). In human heart tissue, ST detected gene expression changes associated with localized cardiomyocyte degeneration (Boogerd et al., 2022). Spatial profiling has also been extended to developmental contexts where studies in monkey and mouse embryos have mapped tissue organization and lineage specification (Cui et al., 2022; Srivatsan et al., 2021), providing potential translational relevance for embryo-fetal development and teratogenicity assessments. Furthermore, integrating scRNA-seq with ST has elucidated the spatial organization and cell composition of bone-marrow niches in mice, revealing region-specific patterns relevant to hematopoietic regulation (Baccin et al., 2020). This is directly aligned with safety questions in developmental and reproductive toxicology, where the location of perturbation can be as important as its magnitude.

A key advantage of spatial omics in toxicology across all tissues and platforms is its ability to strengthen causal understanding by linking tissue structure with molecular mechanisms of response, advancing the molecular pathology field. It is particularly well suited for early hazard identification when changes are focal, for distinguishing primary toxicity from downstream inflammation or repair, and for deriving biomarkers that reflect the anatomic origin of injury. Moreover, combining this with orthogonal layers like histopathology scoring, immunostaining, and scRNA-seq can establish a coherent trajectory from initial site-specific stress responses to multicellular remodeling and eventual adverse outcomes. As spatial technologies mature, these applications are likely to expand from retrospective mechanistic studies into prospective safety workflows, where spatial signatures support compound prioritization, guide follow-up assays, and enable more interpretable cross-species comparisons of tissue injury mechanisms.

**FIGURE 4:**
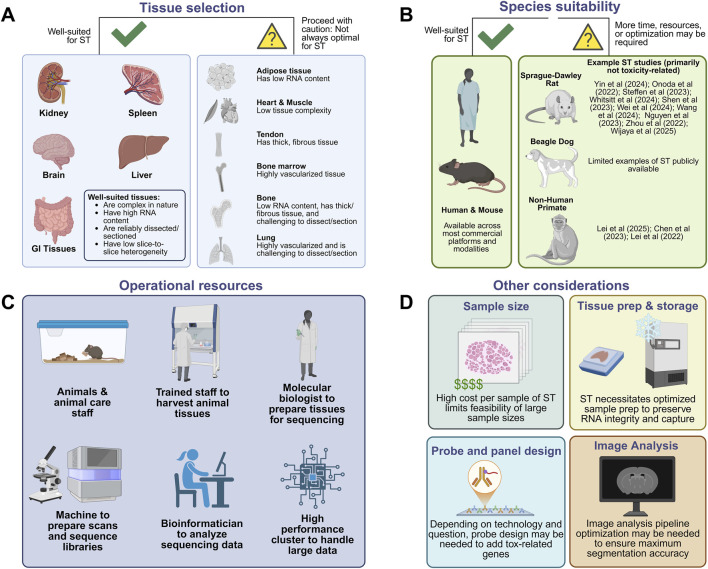
Considerations for spatial platform selection in pharmaceutical preclinical safety studies. **(A)** Tissue types optimal for spatial profiling compared to those presenting technical challenges due to inherent structural or compositional properties. **(B)** Species-specific considerations for spatial profiling, highlighting model organisms with well-established assay availability *versus* those requiring additional optimization, resources, or development time due to limited adaptation for preclinical applications. **(C)** Considerations regarding operational resources required for a successful preclinical study in the pharmaceutical space, including cross-functional teamwork across multiple subject matter experts and proper resources to execute the necessary experiments. **(D)** Other considerations for a successful study, including sample size, tissue preparation and storage, probe and panel design, and image analysis. All figures were generated on an Enterprise license of Biorender. Publication licenses require listing the journal to be published in and expected date of publication. Publishing licenses will be requested upon manuscript acceptance.

The frequent application of ST to the liver and kidney reflects their structural organization and compatibility with current spatial technologies. These organs have well-defined anatomical regions and distinct cellular zonation, making them ideal for high-resolution spatial mapping and mechanistic studies of toxicity. In contrast, some tissues such as the brain, heart, or other tissues may present added challenges for optimization, sample preparation, or data interpretation, resulting in fewer published ST profiles (Figure 4A). As technology advances and protocols become more adaptable, broader use across diverse tissues may become practical. For now, the emphasis on liver and kidney in ST studies highlights their suitability for spatial analysis, serving as benchmark systems for platform validation and development.

## 4 Databases and tools for toxicologists analyzing spatial transcriptomics data

### 4.1 Tissue atlases for toxicology applications

Numerous databases and tools are available for analyzing spatial data, including some comprehensive reviews detailing the tools available for specific computational tasks (Cesaro et al., 2025; Das Adhikari et al., 2024; Gaspard-Boulinc et al., 2025; Yan et al., 2025). Additionally, there are atlases of scRNA-seq and ST data from tissues often used in toxicology, including human kidney atlases in both healthy and diseased states (Chen D. et al., 2023; Lake et al., 2023). For example, Lake and colleagues compiled an atlas of healthy and injured cells in healthy (n = 45) and diseased (n = 48) human kidneys, providing a comprehensive atlas of 51 cell types (Lake et al., 2023). This atlas also provides 28 cellular states in interstitial and nephron segments perturbed in diseased states, including cycling, adaptive, transitioning, and degenerative states. Resources such as these provide references for baseline and perturbed transcriptional states in frequently profiled tissues, providing insight that can be applied to better understand safety flags in preclinical studies. Previous reviews have also catalogued major atlases for other organs, species, and disease models (Zhang L. et al., 2022). Together, these tools and available datasets may also help toxicologists determine the most suitable uses for spatial platforms.

### 4.2 Computational tools for ST analysis

There is a wide range of computational tools for spatial analyses, suited to different project needs (Table 3). The broad selection of tools supports adaptation to various study types, and testing multiple pipelines can help identify the most suitable method. Many artificial intelligence (AI) algorithms are available to uncover insights like spatially variable or predictive genes (Meng-Lin et al., 2023), deconvolve cellular makeup within regions of interest, and explore cell-cell communication based on gene and protein expression (Li et al., 2022). Some algorithms can even infer interactions between cell types by evaluating ligand and receptor expression within nearby cells (Zhang R. et al., 2022). This is useful in toxicity studies, such as mapping drug-induced injury pathways between cell types, like hepatocytes and immune cells in drug-induced liver injury (Ben-Moshe et al., 2022).

**TABLE 3:** Tools relevant for safety analyses with spatial data.

Example methods	Description	Applications in toxicology research
CellChat (Jin et al., 2021), MISTy (Tanevski et al., 2022), Giotto (Chen et al., 2025; Del Rossi et al., 2022; Dries et al., 2021), SpaTalk (Shao et al., 2022), COMMOT (Cang et al., 2023), Cell2cell (Armingol et al., 2022), SpaOTsc (Cang and Nie, 2020)	Identifies cell-to-cell communication using spatial distance and gene expression between different cell types.	Finds interactions between cell populations (i.e., immune and tissue cells).
SCING (Littman et al., 2023), scSAGRN (Ren et al., 2025), SCRIpro (Chang et al., 2024), spGRN (Du et al., 2025), SpaGRN (Li et al., 2025), MultiGATE (Miao et al., 2025)	Maps gene regulatory networks.	Detects key pathways, transcription factors, and drug-sensitive targets within the data.
Cell2location (Kleshchevnikov et al., 2022), Giotto (Chen et al., 2025; Del Rossi et al., 2022; Dries et al., 2021), SPOTLight (Elosua-Bayes et al., 2021), DSTG (Song and Su, 2021), DECLUST (Wang et al., 2025), Redeconve (Zhou et al., 2023), Tangram (Biancalani et al., 2021)	Infers cell types present, their locations, and abundance within tissues.	Reveals which cell populations exist in tissues, even if the data lack single-cell resolution.
BayesSpace (Zhao et al., 2021), SpaGCN (Hu et al., 2021), Giotto (Chen et al., 2025; Del Rossi et al., 2022; Dries et al., 2021), Seurat (Butler et al., 2018; Hao et al., 2024 ; 2021; Satija et al., 2015; Stuart et al., 2019), StLearn (Pham et al., 2023)	Clusters and enhances the resolution of spatial data.	Identifies and describes regions within organs (e.g., immune or nervous tissue).
SpatialDE (Svensson et al., 2018), SPARK (Sun et al., 2020), Giotto (Chen et al., 2025; Del Rossi et al., 2022; Dries et al., 2021), Seurat (Butler et al., 2018; Hao et al., 2024 ; 2021; Satija et al., 2015; Stuart et al., 2019), BayesSpace (Zhao et al., 2021), HEARTSVG (Yuan et al., 2024), BASS (Li and Zhou, 2022)	Finds genes whose expression varies by location in the tissue.	Uncovers how toxicity spreads in tissue based on gene expression patterns across regions.
Giotto (Chen et al., 2025; Del Rossi et al., 2022; Dries et al., 2021), Seurat (Butler et al., 2018; Hao et al., 2024 ; 2021; Satija et al., 2015; Stuart et al., 2019) SquidPy (Palla et al., 2022)	Offers all-in-one analysis for cell-type distribution, spatial gene expression, and cell interaction detection.	Useful for all stages of toxicity data analysis, offering built-in tools for downstream analyses.

One well-known AI tool is MISTy, which can analyze multiplexed spatial omics data and infer cell-cell communication through gene regulatory networks (Tanevski et al., 2022). Using random forest and ensemble machine learning, it infers gene interactions both inside cells and in their surroundings. This helps map regulatory networks at various levels of tissue structure and function. Using breast cancer data from 10x Genomics Visium and Imaging Mass Cytometry, MISTy linked its findings to clinical outcomes, recapitulated known signaling pathways such as estrogen signaling, and highlighted potential novel adjacent molecular interactions.

(Tanevski et al., 2022). It has also been applied to multi-omics datasets in toxicity research, offering more depth than traditional analyses (Nguyen et al., 2022). SCING (Single-cell Integrative Gene regulatory network inference) is another tool, using mutual information and gradient boosting to map drug-related gene pathways and spatial organization (Littman et al., 2023). Of note, these methods were optimized using data from oncology and chronic inflammation (MISTy) or neurodegenerative disease (SCING). As such, it is important to carefully evaluate that they accurately model biological processes commonly present in toxicology settings, including necrosis and fibrosis. If not, it may be necessary to perform transfer learning for proper domain adaptation. As with all findings identified using *in silico* methods, any co-localization or proximity should not be treated as causality. Orthogonal validation should always be leveraged to support key findings and link them back to conventional toxicology readouts (e.g., clinical chemistry, hematology, and histopathology).

More recently, AI-based tools for analyzing spatial omics data have expanded rapidly in variety and scope, enabling greater flexibility to a wider range of research questions. These include Spotiphy, which infers single cell resolution at the whole transcriptome level in sequencing-based ST data using deep learning, validated against high-resolution Xenium and MERFISH imaging from mouse brains and human breast tissues (Yang Jiyuan et al., 2025), and STPath, a generative foundation model that predicts whole transcriptome gene expression using whole slide images paired with ST profiles across 38,984 genes and 17 organs without model tuning, validated on its ability to predict patient survival outcomes and gene mutation status (Huang et al., 2025). While these provide a few examples, the variety of tools is ever-expanding. These examples highlight how computational tools can maximize the downstream value and insights gained from these robust, high-dimensional assays. However, caution should be exercised when interpreting data generated using AI-based tools, and interpretations should consider dose-time dynamics (preferably with pharmacokinetics-informed exposure levels), potential off-target binding, and metabolization of the drug compounds.

### 4.3 Emerging ST technologies and deep learning applications in toxicology

While two-dimensional (2D) spatial reconstructions have advanced our understanding of tissue architecture at the transcriptional and subcellular level in individual slices, these methods remain limited in their ability to provide insights across the third dimension. This third spatial dimension is particularly critical for evaluating complex, spatially heterogeneous, and dynamic tissues such as lymphoid organs, where relying on a single slice fails to capture the full extent of tissue diversity. Recent developments in spatial technologies have improved the feasibility of three-dimensional (3D) tissue reconstructions, thereby opening new avenues for preclinical safety research. Although 3D spatial profiling is not extensively used in toxicology studies, it has shown promising applications in fields like neuroscience for mapping intricate brain regions (Moffitt et al., 2018). The principal advantage of 3D spatial profiling lies in its ability to reveal spatial effects of compounds within cellular microenvironments and gene programs throughout larger tissue volumes—offering valuable mechanistic insights, though current adoption remains constrained by substantial costs and resource requirements.

Three-dimensional spatial profiling can strengthen toxicology studies by capturing continuity, adjacency, and anatomical gradients that 2D slices often miss or distort. Three-dimensional reconstructions reduce sampling bias from section orientation, enable volumetric quantification of lesion burden and connectivity (e.g., confluent *versus* focal injury), and align effects with the microanatomy that governs local exposure. This includes blood flow in the liver, filtrate flow in the kidney, and airflow in the lung. In the liver, 3D mapping clarifies periportal *versus* pericentral injury across entire lobules and reveals how necrosis or inflammation propagates along portal-central axes. In the kidney, following tubules from glomerulus through proximal and distal segments allows segment-specific injury and transport gradients to be measured along continuous structures rather than inferred from isolated cross-sections. In the lung, reconstructing the airway tree dissects patterns of injury, resolving patchy or branching patterns that 2D assays cannot reliably assign. Together, these gains improve mechanistic interpretation, link spatial effects to pharmacokinetics-driven exposure pathways, and provide more accurate endpoints for safety assessments.

While prohibitive costs currently constrain 3D spatial omics applications in toxicology, recent advances in deep learning/artificial intelligence offer promising solutions. Imputation methods using unlabeled histology images can now infer spatial profiles from consecutive tissue sections, streamlining experiment pipelines and enhancing the power to interpret pathology (Bergenstråhle et al., 2022; Monjo et al., 2022). In another example, open-source frameworks like Open-ST enable construction of virtual 3D tissue blocks that integrate histology, gene expression, cell types, and local signaling, potentially improving the ability to model gradients of toxicity (Schott et al., 2024). Other deep learning-driven tools like STMSC improve multi-slice and 3D reconstruction accuracy, potentially helping identify spatial domains affected by toxicity (Zhang et al., 2025). When full volumetric assays are not feasible, multi-slice computational reconstructions such as these can approximate 3D context, provided registration error and uncertainty are quantified and key findings are validated orthogonally. When interpreting results from these methods, one should still be aware of their common limitations, including their dependence on high-quality H&E images and accurate alignment, as misregistration and imaging variability can degrade reconstruction accuracy, and limited generalizability due to batch effects and domain shift across species, protocols, and platforms.

Other advanced computational approaches—including VR-Omics and So3D—offer new ways to analyze, visualize, and interpret toxicological data at high resolution (Bienroth et al., 2025; Zhao et al., 2025). Additional frameworks such as ASIGN, VORTEX, and HoloTea use anatomical and histological data to improve spatial gene expression predictions and consistency across both two and three dimensions, supporting affordable and large-scale toxicology studies (Almagro-Pérez et al., 2025; Sanian et al., 2025; Zhu et al., 2024). Collectively, while findings from these inferences should be interpreted with caution and confirmed with orthogonal validation, recent innovations are driving a new era of spatially defined toxicology. These advances make it possible to extract more value from each experiment and support deeper, more precise mechanistic insights and safety assessments.

## 5 Challenges and considerations with ST for preclinical studies

The adoption of new technologies in toxicology offers significant opportunities but also presents practical challenges. While these methods enable more robust tissue profiling and advance toxicity research, they introduce challenges that require thoughtful planning, particularly in drug development. This is especially important in the pharmaceutical industry, where most assays demand greater cross-functional collaboration and coordination among a larger team compared to similar assays in academic settings. Below, we outline key considerations for implementing spatial profiling within the pharmaceutical sector: tissue selection, species choice, sample size, tissue handling and storage, available resources and expertise, probe and panel design, and image analysis.

### 5.1 Tissue selection

In toxicology studies, an essential step involves identifying where toxicity is occurring and which tissues are affected. Once tissues of interest are selected, researchers can then determine the most appropriate analytical approach. Selecting the right methodology depends largely on whether both the affected tissue and the specific safety question align well with ST capabilities. High-resolution ST offers the most value when investigating lesions or inflammatory responses involving multiple interacting cell types, regions with active immune responses, or cases where the source or extent of toxicity is unclear, such as focal toxicity in gastrointestinal tissue or of the biliary tract in the liver. For widespread or uniform tissue damage, or situations dominated by necrosis (which reduces the quality of RNA), bulk RNA-seq is generally more suitable and efficient. When toxicity affects a limited number of cell types, or in tissues with diverse cellular composition like the kidney or immune organs, scRNA-seq may be preferable.

The selection of appropriate spatial transcriptomics platforms for toxicity studies requires careful consideration of tissue-specific architecture and resolution requirements (Figure 4A). Some tissues, such as bone marrow or tendon, are difficult to section for FFPE assays. Others, such as fat, bone, and lung, have low RNA content or cell density, reducing ST’s effectiveness. In cases where profiling these tissue types is a priority, additional steps may be needed, i.e., planning additional lead time to decalcify bone samples before sequencing preparation. Alternatively, if ST is not essential, LCM or scRNA-seq are reasonable alternatives for these sample types. Highly vascular or lymphoid tissues (e.g., lung, lymph node, spleen, thymus) contain rapidly changing cell populations, making it challenging to capture relevant interactions within a single section. Tissues with uniform cell types (e.g., cardiac or skeletal muscle) typically warrant ST profiling only in regions of known pathology, such as heart valves or conduction tissue. However, profiling localized heart regions requires specific slide preparation to ensure the region of interest is available, and may only provide valuable insights if arrhythmia, abnormal ECGs, or other forms of stress are observed in-life. For more diffuse cardiac pathologies, bulk RNA-seq or LCM provide sufficient resolution to discern underlying toxicities. Finally, when toxicity affects multiple organs, those with greater cellular heterogeneity (like kidney, liver, intestine, endocrine, reproductive tissues, and skin) often provide more informative data with ST. These factors should be systematically considered before selecting a ST platform and tissue.

Ultimately, successful tissue profiling with ST depends on three factors: feasibility of profiling the tissue type, compatibility of the tissue and injury type with the chosen ST platform, and availability of and access to the region of interest for localized lesions. Then before proceeding with ST for a toxicity study, it is important to consider whether ST is justified over another assay with lower demand for resources.

### 5.2 Species suitability

Species selection is critical in preclinical toxicology studies utilizing spatial profiling (Figure 4B). In drug development, the most used species for safety studies include rats, dogs, and monkeys, with limited use of mouse or human models (Prior et al., 2018). Most commercial ST platforms are optimized for mouse or human tissue, which limits their immediate applicability to commonly used preclinical species. While there are examples of successful safety-related ST applications in rat (Onoda et al., 2022; Wijaya et al., 2025), these remain uncommon, with even fewer examples in dogs and monkeys (Table 4). Expanding beyond the scope of safety, there are additional studies that have used spatial platforms to profile rats (Nguyen et al., 2023; Shen et al., 2023; Steffen et al., 2023; Wang et al., 2024; Wei et al., 2024; Whitsitt et al., 2024; Yin et al., 2024; Zhou et al., 2022) and monkeys (Chen A. et al., 2023; Lei et al., 2022; 2025), providing examples of how these technologies can be leveraged in less common preclinical models. The scarcity of these studies in non-murine, non-human species is often due to the limited availability of validated assays and probe panels for these species.

**TABLE 4:** Case studies of spatial profiling in safety-related studies.

Author/Journal	Tissue	ST platform	Species	Summary of findings
Nault et al. (2023), *Toxicological Sciences* (Nault et al., 2023)	Liver	Molecular Cartography (Resolve Biosciences)	Mouse	TCDD exposure causes dose dependent disruption of hepatic zonation, with loss of portal identity and Wnt/βcatenin signaling disruption.
Onoda et al. (2022), *DNA Research* (Onoda et al., 2022)	Kidney	10x Visium	Sprague-Dawley Rat	Losartan induces upregulation of renin pathway genes, with glomerular spatial heterogeneity in response.
Xin et al. (2023), *Commun Biol* (Xin et al., 2023)	Liver	NanoString GeoMx DSP	Mouse	Pericentral hepatic zones showed the most I/R damage, with spatially defined immune infiltration and hepatocyte injury signatures.
Jiang et al. (2024), Am J Pathol (Jiang et al., 2024)	Liver	10x Visium	Mouse	WD + CCl_4_ exposure induced periportal CYP2E1 and lipid metabolism gene enrichment, mimicking pediatric MASH.
Zhou et al. (2024), *Metabolism* (Zhou et al., 2024)	Liver	10x Visium	Mouse	RSPO3 and other Wnt modulators became ectopically expressed across lobule zones, disrupting normal liver zonation.
Xu et al. (2024), *Nutrients* (Xu et al., 2024)	Brain	10x Visium	Mouse	High maternal folic acid consumption affects neurogenesis, neuronal axon myelination pathways, and gene expression linked to learning and memory in thalamic and ventricular regions.
Wijaya et al. (2025), *Cell Biol Toxicol* (Wijaya et al., 2025)	Kidney (Nephron)	LCM + TempO-Seq	Sprague-Dawley rat	Identified region-specific DNA damage, inflammatory signaling, actin cytoskeletal remodeling and increased glycolytic metabolism in multiple distinct regions of the kidney.

Platforms that accept flash-frozen samples such as 10x Genomics Visium HD, 10x Genomics Xenium, NanoString GeoMx DSP, NanoString CosMx SMI, and Akoya Phenocycler can increase flexibility and facilitate research in non-traditional species. Despite these advances, a practical challenge remains: many preclinical and archival studies do not have flash-frozen tissue regularly collected nor readily available, which restricts the use of these newer, more flexible technologies. Assay development (e.g., custom probe panels, pilot studies) may be necessary when extending spatial profiling to new species, which can increase time and resource requirements (Figure 4C).

Most spatial omics panels are designed primarily for mouse or human tissues, so adapting these technologies to preclinical models often necessitates the development of custom panels, extensive in-house testing, and further optimization of laboratory and analytical workflows (Figure 4D). When creating custom panels, it is essential to incorporate both toxicity-related genes and markers tailored to the relevant cell types. Care should be taken to avoid overcrowding probes in compact tissue regions, since excessive probe density can cause optical crowding and signal overlap, complicating interpretation (Moses and Pachter, 2022). It is important to strike a balance between panel size and sensitivity to reliably detect both common and rare targets. Throughout development and implementation, rigorous validation and comprehensive external quality checks are important to ensure robust, interpretable results.

In summary, while cross-species spatial profiling is becoming more feasible due to emerging technologies and ongoing development efforts, mouse and human tissues continue to dominate the field due to platform compatibility and reagent availability. Researchers should consider these practical limitations early in study design and consult with ST technology providers regarding available options or custom solutions for their species of interest.

### 5.3 Operational resources

Large spatial studies rely on highly skilled, multidisciplinary teams and strong communication among all members (Figure 4C). Core contributors typically include animal model experts, pathologists, histologists, molecular biologists, and bioinformaticians, with technical specialists or vendor representatives providing support as needed depending on the technology platform. Key resources such as specialized reagents, high-performance instrumentations, high-resolution imaging and scanning microscopes, and robust computational infrastructure are also essential for successful experiments. Access to adequately powered, age- and sex-matched animal cohorts and dedicated husbandry support for dosing and timely tissue collection are critical. To ensure consistent adherence to protocols, all team members must be informed of study timelines, given regular updates, and made aware of any technical considerations in tissue preparation and storage, as lapses in timely communication can lead to study deviations or delays.

Applying the considerations outlined above in particular for selection of appropriate types of lesions, tissues, and mechanistic toxicology questions requires clear communication between pathologists and other scientists on these cross-functional teams. Not all lesion types require leveraging complex and expensive ST techniques to investigate the source of toxicity (i.e., gastrointestinal crypt necrosis with radiomimetic agents). While others may warrant careful consideration of ROI selection (i.e., immune infiltrates in liver with immune-oncology agents). For facilities that may not have access to close collaboration with pathologists, these considerations can pose a significant challenge.

During study preparation, it is vital that histologists and molecular biologists deliver high-quality tissue scans and comprehensive metadata for bioinformatics analysis. These teams, together with bioinformaticians, need to communicate clearly regarding sample IDs, treatment groups, and any upstream issues that could influence data analysis. Notable findings affecting downstream processing should also be promptly shared. Ultimately, robust teamwork and clear communication are essential to meeting deadlines and achieving reliable outcomes, particularly in the pharmaceutical preclinical safety space, where minor communication gaps can quickly become significant study concerns.

### 5.4 Sample size and tissue preparation and storage

Sample size and tissue preparation are also key considerations for a successful preclinical safety study (Figure 4D). Most spatial studies focus on mapping gene expression in healthy or diseased tissues (Lake et al., 2023; Zhang R. et al., 2022). To apply spatial methods to toxicology studies in a longitudinal manner, compound dose (i.e., potency) and timing (i.e., pharmacokinetic or pharmacodynamic data) should be considered. For instance, drug-induced liver injury can have many possible responses depending on dose, as different pathways are switched on or off (Dara et al., 2017; Wink et al., 2018). This means that sampling across multiple doses and time points would be beneficial but increases cost and logistical complexity. Curating large enough sample sizes with quality tissue can also be challenging—older tissues may have degraded RNA, or storage differences can introduce technical variability. As ST assay sensitivity improves, small technical artifacts may become more pronounced. A typical exploratory toxicity study suitable for ST sample collection usually involves 3 rodents for each of 3 escalating doses, totaling 12 animals per sex including controls. Not all dose groups require analysis; selection often depends on histological findings or tolerability, which helps streamline workload and costs. For large animal studies, sample sizes are usually smaller (1–2 animals per group), while targeted studies may justify larger groups at established doses if needed. Regardless, the complexity of profiling multiple drug doses across time points adds significant hurdles to spatial studies in the pharmaceutical space due to monetary, workload, and tissue availability limitations. These hurdles may be further compounded by variability in the stored tissue needed to conduct these studies, therefore planning in advance for ST profiling may be beneficial when feasible.

### 5.5 Image analysis

Accurate image analysis is essential for reliably mapping transcriptomic data to tissue morphology in studies leveraging spatial platforms (Figure 4D). Fundamental steps include robust image alignment, registration, and the standardization of scan quality and metadata, which are particularly critical when comparing results across multiple groups. Requesting an expert pathologist’s review of the study’s histological findings can provide key insights into relevant tissue structures prior to any computational analysis. In addition, computational quality control is necessary to identify and remediate artifacts such as poor focus, tissue tears, staining anomalies, or problematic spots and markers, ensuring data integrity throughout the study.

Building upon rigorous image preprocessing, advanced image segmentation can often be achieved using deep learning tools [e.g., Cellpose (Stringer et al., 2021), Mesmer (Greenwald et al., 2022), StarDist (Stevens et al., 2022), UNet++ (Zhou et al., 2018), Instanseg (Goldsborough et al., 2024)]. These can be resource-intensive and should be chosen based on the dataset’s specific characteristics. It is important to validate segmentation results by benchmarking against pathologist assessments. Subsequent modeling steps [e.g., using SpatialDE (Svensson et al., 2018)] are sensitive to noise in image or coordinate data, which can add complexity to the analysis. For all computational findings, orthogonal validation and expert review are essential to ensure accuracy and reliability.

## 6 Future directions of spatial transcriptomics in toxicology

### 6.1 Improved assay robustness and sensitivity

ST technologies are continually improving, addressing limitations in earlier platforms such as 10x Genomics Visium (Ståhl et al., 2016), NanoString CosMx Spatial Molecular Imager (SMI) (He et al., 2022), and Akoya Phenocycler (formerly CODEX) (Heinrich et al., 2022). New array-based assays deliver higher spot resolution, expanded gene coverage, improved tissue compatibility, and integrated analytics that combine gene expression and tissue morphology from a single section.

Imaging-based platforms have also advanced, offering faster workflows, larger panels for RNA and protein targets, and more marker options, including those tailored for specific fields like immuno-oncology and neurology. Many of these updated platforms can now support both single-cell and spatial profiling simultaneously, reducing reliance on separate scRNA-seq experiments to capture cellular diversity and tissue structure. Increasingly, single-cell spatial transcriptomics datasets also incorporate protein and morphological data from the same slide, enabling more comprehensive and robust analysis from a single tissue sample.

These advancements in assay technology are particularly valuable in preclinical safety, where animal studies often involve significant costs and limited tissue availability. By enabling the collection of multiple types of data from a single tissue section, researchers can conserve precious samples and reserve additional material for other analyses, thereby enhancing the overall strength and reliability of toxicological findings.

### 6.2 Increased accessibility of spatial omics platforms

As spatial omics technologies such as transcriptomics, proteomics, and imaging become more widely adopted, the introduction of new platforms from independent vendors is expanding the field. This growing market offers several key advantages: it drives down costs, accelerates innovation in features and capabilities, and provides researchers with more choices suited to a variety of scientific questions and model organisms. For example, the Standard Biotools (formerly Fluidigm) Hyperion Imaging System can detect up to 40 protein markers simultaneously in tissue using mass cytometry combined with imaging (Chang et al., 2016). Vizgen’s MERSCOPE, using MERFISH technology, delivers subcellular spatial resolution and enables analysis of up to 500 RNA targets in a single sample (Moffitt et al., 2016). As additional platforms become available, many limitations of earlier assays are being resolved. Importantly, these advanced technologies increasingly support both single-cell and spatial profiling in the same workflow, reducing the need for separate experiments and enabling more comprehensive tissue analysis.

### 6.3 Potential applications of spatial omics in modeling and simulation paradigm

Model-informed Drug Discovery and Development (MID3) approaches to assess drug-induced organ toxicity such as Quantitative Systems Toxicology (QST) have gained more attention in the pharmaceuticals space in recent years. The QST approach demonstrated values both for supporting internal decision making to select and advance best drug candidates as well as for supporting regulatory submissions (Ferreira et al., 2020; Goldring et al., 2025). An advanced QST modeling platform such as DILIsym was successfully applied to investigate the clinical risk and underlying mechanisms of hepatotoxicity for several drugs reported to cause drug-induced liver injury in clinical studies (Watkins, 2020).

Spatial omics holds great potential values to inform QST models, not only on the mechanisms of toxicity, but also on the site of occurrence as well as the interactions among cells and tissues. This leads to QST models with a comprehensive representation of organs’ pathophysiology that enables simulating and predicting toxicity outcomes through space and time. Recent studies using QST modeling of individual intestinal crypt cells have revealed distinct patterns of cell-to-cell signaling and communication that modulate cell fate and differentiation (Gall et al., 2023). Another recent ST profiling study that identified differential deregulation across nephron segments following chemotherapeutic treatment could also inform QST model development for studying the functional and spatial mechanisms of drug-induced kidney injury (Wijaya et al., 2025).

## 7 Conclusion

Spatial transcriptomics provides a step change in preclinical safety assessment because it links gene expression to tissue structure. This spatial context can be decisive when toxicity is focal, zonated, or confined to specific tissue compartments, and when interpretation depends on aligning molecular changes with histopathology. In these situations, ST can increase mechanistic confidence and reduce ambiguity that can occur with bulk profiling alone. ST should not be, however, an automatic default for every study. In drug development, designs typically span multiple doses, durations, and time points, and the experimental and analytical workload increases with each added group. The added effort is justified when spatial resolution is required to answer the primary question. When it is not, conventional approaches often remain the most efficient route to an actionable conclusion. Similarly, tissue types less amenable to ST profiling but commonly profiled in toxicology studies will require additional steps, preparation, or optimization to ensure satisfactory assay performance. If an alternative assay type such as bulk RNA-seq, LCM, IHC/IF, or scRNA-seq can provide sufficient insights for the given study, this is often desirable given the resource-intensive nature of ST. For example, if toxicity is suspected in the heart’s conduction system in a dog study, LCM or bulk RNA-seq provide an effective alternative to ST. Both methods precisely profile the primary site of pathology while requiring significantly less optimization and fewer resources given the limited nature of dog panels across ST platforms.

Experience with transcriptomic decision-support tools demonstrates the importance of thoughtful implementation and positioning. Research applications of bulk liver transcriptomics have shown clear value for risk assessment and study prioritization, yet these tools work best as part of a comprehensive evaluation framework rather than as standalone decision-making platforms. Through extensive use across multiple studies, both strengths and limitations become apparent, including characteristic patterns of false positives and negatives that require careful interpretation.

Practical challenges can significantly impact the long-term utility of these approaches. This became particularly evident during the technological transition from microarray to RNA-sequencing platforms, when shifts in profiling technologies introduced substantial differences in data annotation and quantitative behavior. These platform changes disrupted established scoring systems and created continuity issues that hindered consistent application. Additionally, when increased costs and operational complexity associated with newer sequencing technologies limited routine implementation, it became more difficult to accumulate the consistent datasets needed for ongoing refinement and validation of these tools. This experience underscores the need for sustainable, well-integrated approaches to transcriptomic decision support that account for both technical evolution and operational considerations, particularly during periods of rapid technological advancement.

Spatial transcriptomics is a powerful technology, but it introduces its own assumptions and failure modes, and confidence depends on repeated, disciplined use. Adopting this technology for toxicity studies is not trivial and often carries with it limitations in supported model species, significant lead time for optimizing custom panels or challenging tissue types, efficient cross-discipline collaboration, and analysis and storage of large amounts of data. In the short term, the highest impact for spatial transcriptomics in toxicology is likely to be mechanistic and interpretive, particularly when spatial context clarifies lesion biology and supports translatability. Over the longer term, sustained value will require standardization, integration with complementary assays, and deliberate choices about when ST is essential *versus* when simpler approaches are sufficient. Used in this way, ST can strengthen safety decisions and contribute to more efficient drug development without displacing methods that remain fit for purpose.
